# Sweet Taste Adaptation to Sugars, Sucralose, and Their Blends: A Human and Rodent Perspective

**DOI:** 10.3390/nu17193075

**Published:** 2025-09-27

**Authors:** Stephanie I. Okoye, Minjae Kim, Sara Petty, Myunghwan Choi, Marta Yanina Pepino

**Affiliations:** 1 Division of Nutritional Sciences, University of Illinois Urbana-Champaign, Urbana, IL 61801, USA; siokoye2@illinois.edu; 2 School of Biological Sciences, Seoul National University, Seoul 08826, Republic of Koreachoim@snu.ac.kr (M.C.); 3 The Institute of Molecular Biology and Genetics, Seoul 08826, Republic of Korea; 4 Food Science and Human Nutrition, University of Illinois Urbana-Champaign, Urbana, IL 61801, USA; saraap2@illinois.edu; 5 Carle Illinois College of Medicine, Urbana, IL 61801, USA

**Keywords:** psychophysics, taste buds, calcium imaging, cross-adaptation, LCS consumers

## Abstract

**Background:** Sweet taste adaptation, the decline in perceived sweetness with repeated exposure, may influence dietary behavior and differs across sweeteners. Low-calorie sweeteners (LCSs) such as sucralose strongly activate the T1R2+T1R3 receptor and are generally associated with greater adaptation than sugars, although this effect can be reduced with sweetener blends. **Aim:** We investigated whether habitual LCS consumption affects sweet taste perception and whether blending sucralose with small amounts of sugars attenuates adaptation using sensory tests in humans and in vivo calcium imaging in a rodent model. **Methods:** In study 1, habitual (HC; *n* = 39) and non-habitual (NHC; *n* = 42) LCS consumers rate sweetness of sucralose (0.6 mM), glucose (800 mM), fructose (475 mM), and blends with low glucose (111 mM) or fructose (45 mM) across repeated trials (1–10) using a generalized labeled magnitude scale. In study 2, a microfluidic-based intravital tongue imaging system was used to assess in vivo responses to sweet adaptation in genetically modified C57BL/6 mice (*n* = 8) expressing a calcium indicator in type II/III cells of taste buds. **Results:** Habitual LCS use was not associated with differences in sweetness perception or adaptation (all *p*-values > 0.6). Sucralose alone produced stronger adaptation than when blended with sugars in both humans (*p*-values < 0.002) and mice (*p* < 0.001). Glucose and fructose alone showed adaptation (relative decrease reached on final trial compared to the first trial: −27% ± 4% for glucose, −38% ± 5% for fructose, both *p*-values < 0.002) but to a lower degree compared with sucralose (−66% ± 5%). **Conclusions:** Sweetener composition, rather than habitual LCS use, drives sweet taste adaptation. Blending sucralose with small amounts of sugars reduces adaptation at both perceptual and cellular levels, providing mechanistic insights relevant to the formulation of LCS products.

## 1. Introduction

Recent structural and functional analyses of the human sweet taste receptor (T1R2 + T1R3) have deepened our understanding of how diverse sweet-tasting compounds activate the receptor to elicit sweetness perception. For example, Juen et al. [1] demonstrated that structurally distinct sweeteners, such as sucralose, a widely consumed low-calorie sweetener (LCS), and sucrose, a natural caloric sugar, bind to a common pocket on the T1R2 subunit. This binding initiates G protein signaling and activates the sweet taste pathway [1]. Complementary calcium imaging confirmed that all tested sweeteners activate this receptor complex, though with varying affinities, which aligned with their perceptual sweetness [1]. Notably, sucralose, which is approximately 600 times sweeter than sucrose, elicited more efficient receptor activation than the caloric sugar [1].

However, the pharmacology of the T1R2 + T1R3 heterodimer is complex, not only because the receptor presents multiple binding sites capable of accommodating ligands with different chemical structures, but also because a single ligand can interact with more than one site, although with different affinities [2,3]. Some binding sites directly mediate conformational changes that activate the receptor, whereas others contribute to allosteric modulation (either positive or negative). Positive allosteric modulators, such as SE-1 and SE-2, increase receptor sensitivity [4], while negative allosteric modulators, such as lactisole, decrease receptor activation [5]. These findings underscore that while sweeteners may share common binding mechanisms, their structural differences result in distinct functional responses—emphasizing the need to consider individual sweeteners, rather than treating them as a single class in research on individual differences in sweetness perception, including sensory adaptation.

Sensory adaptation, defined as a decrease in perceived intensity following repeated or sustained stimulation, is a well-established phenomenon across sensory systems [6]. In the context of taste, adaptation is particularly important because it can influence satiety-related cues. For example, changes in peripheral sweet taste signaling have been linked to alterations in food acceptance, satiety hormone release, and central appetite processing, all of which may affect overall energy intake and post-meal satisfaction [7]. Classic studies of taste adaptation have highlighted considerable interindividual variability influenced by stimulus concentration, exposure duration, oral movements, temperature, and salivary properties [8,9,10,11]. However, the potential role of dietary influences on sweet taste adaptation, particularly the frequent exposure to sweetness from potent LCS, remains largely unexplored.

Adaptation has been observed for both caloric and non-caloric sweeteners [12]. At the molecular level, evidence suggests that sweet taste adaptation primarily involves peripheral mechanisms in type II taste cells, which express G protein-coupled receptors (GPCRs), and glia-like type I cells [13,14]. Repeated stimulation of GPCRs, such as the T1R2 + T1R3 heterodimer, leads to kinase-mediated phosphorylation receptor desensitization and activation of adjacent glia-like cells, ultimately dampening sweet signal transmission and receptor internalization [14]. Sensory studies in humans indicate that sweet adaptation is more pronounced with LCSs like sucralose than with sugars [15,16,17]. Because LCS can elicit stronger receptor activation than sugars [1], habitual LCS consumption may promote receptor desensitization, diminished sweet taste intensity perception, and accelerate sweet taste adaptation over time. Blending sweeteners may attenuate this effect. Bornstein [18] reported that LCS–sugar mixtures adapted less than LCS–LCS blends. Other studies likewise found reduced adaptation with 50:50 LCS–sugar blends compared with LCS alone [15,16,17]. Although blends of LCSs with sugars are often used in the food industry for bulking, it remains possible that even small amounts of sugar may improve the sweetness profile.

The current study integrates complementary human and animal experiments to investigate sweet taste adaptation. First, we assessed perceptual responses to caloric sweeteners (glucose and fructose), sucralose, and their mixtures using a ratio designed to emulate commercial formulations. We compared perceptual responses in habitual and non-habitual LCS consumers and hypothesize that compared to non-habitual LCS consumers (NHCs), habitual consumers (HCs) would show reduced sweetness intensity perception and greater adaptation across repeated exposures. We also hypothesized that barely detectable amounts of sugars, such as those included for bulking, reduce adaptation to the sweetness of sucralose. Second, to complement the psychophysical data, we employed microfluidics-on-a-tongue (µTongue) calcium imaging in a mouse model to monitor real-time functional activity of taste cells when repetitively stimulated with sucralose, and its binary mixture with glucose and fructose. Here, we hypothesized that the presence of small amounts of sugar, like those used in the sensory study in humans, would attenuate the decrease in calcium responses that would take place when taste buds were repetitively stimulated with sucralose alone. Together, these complementary approaches allow us to examine sweet taste adaptation at both the perceptual and cellular levels, providing a framework to explore how different sweeteners shape sensory responses.

## 2. Materials and Methods

### 2.1. Study 1: Human Psychophysics Study Methods

#### 2.1.1. Participants

A total of 100 adult participants were recruited from the University of Illinois Urbana-Champaign (UIUC) campus between February 2019 and September 2023. No sensory testing was conducted from March 2020 to December 2021 due to COVID-19 restrictions.

Participant eligibility was assessed through a phone or online screening questionnaire. Individuals were eligible if they were between the ages of 18 and 64 years old and reported consuming either more than five LCS-containing products per week (HC) or fewer than one per week (NHC). For the purposes of classification, one LCS-containing product was defined as any item with an amount of LCS equivalent to that in a 12-ounce can of diet soda. To estimate weekly intake, volunteers were asked detailed questions about their current eating habits, including the frequency and quantity of LCS-sweetened foods and beverages consumed. These screening results were later confirmed during the study visits using a non-nutritive sweetener food frequency questionnaire (NNS-FFQ) [19]. Participants who reported consuming between one and five LCS products per week were excluded.

**Figure 1:**
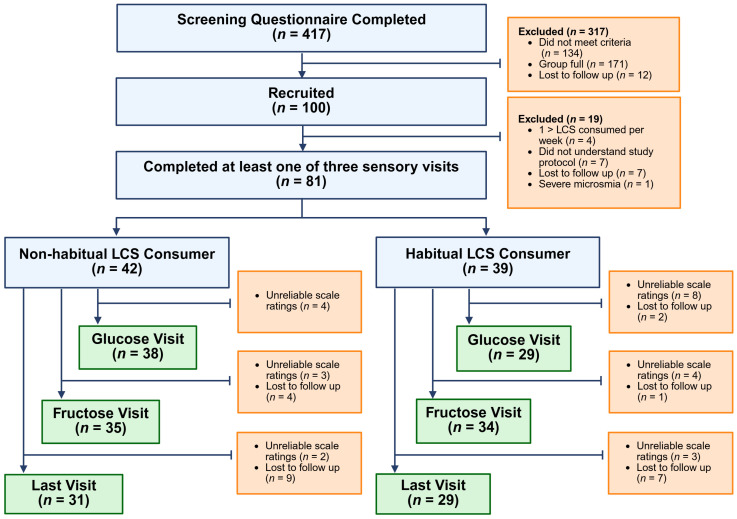
Participant recruitment. Data were excluded if participants’ ratings failed to meet the predetermined criteria described in Section 2.1.3.

Additional exclusion criteria were factors known to affect taste perception (such as current smoking or smoking cessation within six months prior to the study, diabetes, gastric surgery, pregnancy, or severe impairment of smell) and factors that could prevent adherence to the study protocol (e.g., dietary restrictions like fructose intolerance) [20,21,22]. After sensory testing resumed post-pandemic, participants were also asked to complete the University of Pennsylvania Smell Identification Test (UPSIT), and individuals scoring in the severe microsmia range were excluded [23]. See Figure 1 for a breakdown of participants included in the study.

All participants who were recruited provided written informed consent, and the study protocols were approved by the Institutional Review Board at the University of Illinois Urbana-Champaign (IRB #19325).

#### 2.1.2. Procedure

All eligible participants were invited to complete three sensory visits. In preparation for each visit, participants were asked to fast for one hour prior. To calculate BMI, their weight and height were measured at the beginning of the first visit. Sensory visits included taste intensity and adaptation testing with solutions containing sucralose, glucose, fructose, or binary blends of sucralose with one of the simple sugars. Sensory visits also included a taste preference test and several eating behavior questionnaires that will be reported elsewhere. The first two sensory visits, referred to as the glucose visit and fructose visit, were randomized and named based on the simple sugar included in the solutions tested during each session. The third visit, referred to as the last visit, remained constant and had sensory tests involving both sugars tested. Further details on solution preparation and sensory procedures are provided in Section 2.1.4, Section 2.1.5 and Section 2.1.6.

#### 2.1.3. Sensory Assessments Using the General Labeled Magnitude Scale

Participants rated the perceived taste intensity of each solution using a computerized version of the generalized labeled magnitude scale (gLMS), a validated psychophysical tool for measuring perceived intensity [24]. After each sample tasting, they used the Compusense platform (Compusense Inc., Guelph, ON, Canada) to first identify which basic taste qualities they perceived, selecting from sweet, salty, sour, savory, bitter, or no sensation. For each selected quality, they then rated the intensity using separate gLMS scales. The gLMS displays verbal descriptors to participants that correspond to numerical values that are used for statistical analysis: No sensation (0), Barely detectable (1.4), Weak (5.8), Moderate (16), Strong (35), Very strong (53), and Strongest sensation of any kind (100) [24].

Participants received training on how to use the scale at the start of their first visit. Data were excluded if their responses when first tasting sugars in the adaption test were perceived as “no sensation” in the sweet scale.

#### 2.1.4. Sensory Stimuli

Fructose and glucose were obtained from Acros Organics (Fair Lawn, NJ, USA), and sucralose from Spectrum Chemical (Gardena, CA, USA). Details on the content of each solution tested are presented in Table 1. To determine appropriate testing concentrations, we employed several strategies. First, we aimed to match the sweetness intensity of a standard soft drink, which is typically rated around “moderate” on the gLMS. This level was chosen to ensure a sufficient dynamic range to detect potential differences in sweet taste perception between HCs and NHCs. We estimated that this sweetness corresponds to approximately five packets of a commercially available sucralose-based sweetener dissolved in 250 mL (about 8 ounces) of water. Each available commercial packet contains approximately 12 mg of sucralose and 988 mg of glucose, yielding concentrations equivalent to 0.6 mM of pure sucralose and 111 mM glucose [25]. These concentrations were used for the first binary blend. An 800 mM glucose solution was included as an equi-sweet match to 0.6 mM sucralose based on previous research [21] and pilot testing. Additionally, fructose solutions of 475 mM and 45 mM were selected to be equi-sweet to the 800 mM and 111 mM glucose solutions, respectively, based on prior studies [26,27] and preliminary sensory testing.

All samples were prepared using deionized (DI) water and presented as clear solutions in 10 mL aliquots at room temperature.

#### 2.1.5. Taste Intensity Test

To examine potential differences in sweet taste perception related to habitual LCS consumption, participants completed a series of taste intensity tests. The solutions tested varied across the study visits. In the glucose visit, solutions tested included sucralose 0.6 mM, GLU 800, GLU 111, and Sucralose + GLU 111, while in the fructose visit, solutions tested were sucralose 0.6 mM, FRU 475, FRU 45, and sucralose + FRU 45 (Table 1).

Solutions were presented in randomized order, using the sip-and-spit method. Participants swirled each solution in their mouth for 5 s, then expectorated and rated the intensity of any perceived taste qualities as described in Section 2.1.2. Participants rinsed their mouths three times with deionized water between samples, and samples were presented every 60 s. The test was conducted twice per visit, with a 3 min break between sets.

#### 2.1.6. Sweet Taste Adaptation

During each sensory visit, participants completed two sweet taste adaptation tests. Each test consisted of a series of 10 sweet solutions delivered at 15 s intervals, without mouth rinsing between samples. Participants rated each solution using the same procedure as in the taste intensity test.

The specific solutions varied across study visits. In the glucose visit, each test series began and ended with a 10 mL sample of GLU 800. The eight intermediate samples (trials 2–9) served as adapting solutions. One series used sucralose alone, while the other used a binary mixture of sucralose + GLU 111. The order of these two series was randomized. In the fructose visit, the test series began and ended with FRU 475, with adaptation trials using either sucralose alone or a binary mixture of sucralose + FRU 45.

**Figure 2:**
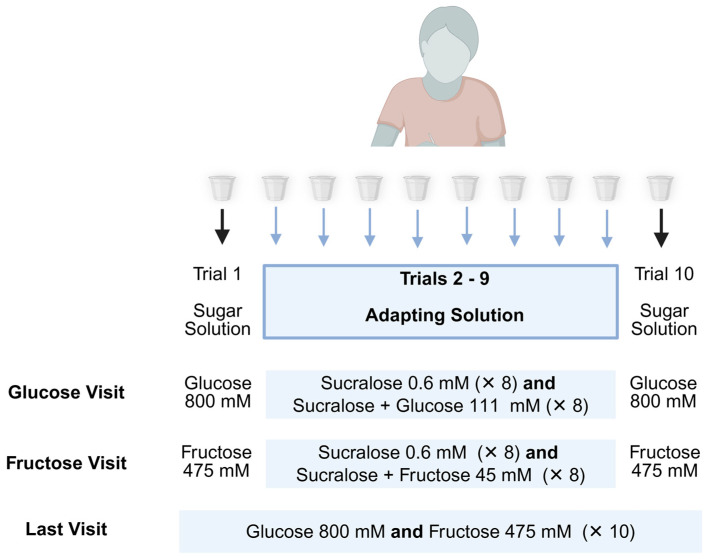
Adaptation Sequence.

During the final visit, participants completed two series composed entirely of either 10 samples of GLU 800 or 10 samples of FRU 475, to assess differences between LCS groups when adapting to simple sugars. The order of adapting solutions was randomized for each participant within each visit. The full sequence of solutions for each test is shown in Figure 2.

After completing the first adaptation series, participants performed a standardized palate cleansing: rinsing with deionized water three times, eating 1–2 unsalted crackers (Kellogg’s, Mississauga, ON, Canada), rinsing again three times, and resting for at least three minutes before beginning the next series.

### 2.2. Study 2: In Vivo Calcium Imaging During Sweet Adaptation in a Mouse Model

To complement the human sensory data with mechanistic insight at the receptor level, we conducted a second study using in vivo calcium imaging in a mouse model. In this model, mice are genetically modified to express calcium indicators in type II/III cells and afferent nerves of taste buds [14]. This allowed us to directly assess sweet taste receptor activity during repeated exposure to sweeteners, under controlled experimental conditions not feasible in human subjects.

This rodent study was conducted at Seoul National University, and all procedures adhered to institutional guidelines and were approved by the Subcommittee on Research Animal Care at Seoul National University (protocol code: SNU-24612-2).

#### 2.2.1. Mouse Preparation and Housing

In this study, Pirt-GCaMP6f-tdTomato mice were used and were bred by crossing PIRT-Cre mice (provided by Xinzhong Dong, Johns Hopkins University) with CaG-floxed-GCaMP6f-tdTomato mice (#031968, Jackson Laboratory). The mice were housed in groups of two to five under a reversed 12:12 h light/dark cycle, with food and water available ad libitum, and were maintained at optimal conditions (22–23 °C, 40–60% humidity). A total of eight 8-12-week-old mice (3 males and 5 females) housed in 5 separate cages were used, and no significant sex-related differences were observed.

#### 2.2.2. Imaging Process & Tastants

In vivo imaging was conducted using the μTongue method as previously described [14,28]. Mice were anesthetized with ketamine (100 mg/kg) and dexmedetomidine (1 mg/kg) given via intraperitoneal injection. Anesthesia was confirmed via toe pinch. The scalp and periosteum were then removed using surgical scissors and cotton swabs. A custom head fixer was attached to the exposed cranium using super glue and dental resin to minimize movement during imaging. The ventral part of the tongue and lower tip were affixed to the microfluidic device with medical adhesive, while the dorsal part of the tongue was immersed in artificial saliva during the imaging preparation.

Mice were then mounted under an upright spinning disk confocal microscope, and artificial saliva was delivered for 20 s as a baseline, followed by tastants (sucralose 2.4 mM or mixtures) applied for 5 s in 10 repetitions with 15 s intervals of artificial saliva to assess adaptation. An 8-channel fluid delivery system (Octaflow II, ALA Scientific, East Farmingdale, NY, USA) connected to an 8-port manifold (MPP-8, Harvard Apparatus, Holliston, MA, USA) was used to deliver tastes, and a syringe pump was used to maintain a flow rate of 300 μL/min. Tests with the different series of tastants were separated by at least 3 min to minimize crosstalk between stimuli.

We initially attempted to induce a response with 0.6 mM sucralose to use the same concentration used in the human studies; however, the method failed to produce an image of a stable calcium response at this concentration (the figure in Section 3.1.4). Subsequently, we incrementally increased sucralose concentrations and found that a minimum concentration of 2.4 mM was required to elicit a sustained elevation in calcium response (the figure in Section 3.1.4). We then selected the 2.4 mM sucralose to study adaptation to blends containing sucralose in combination with either 45 mM fructose or 111 mM glucose. To assess adaptation to each solution, ten 5 s stimulations were delivered with 15 s intervals. All solution types were presented in a randomized order, and all solutions were prepared in the artificial saliva (composition: 2 mM NaCl, 5 mM KCl, 3 mM NaHCO_3_, and others, pH 7.4–7.6).

For imaging, we used a spinning disk confocal unit (CSU-W1, Yokogawa, Musashino, Japan) coupled with a dual sCMOS camera (Prime 586 BSI, Teledyne Photometrics), laser system (iChrome MLE, Toptica Photonics, Gräfelfing, Germany, wavelength: 405, 488, 532, 640 nm), and a motorized upright microscope (Ni-U, Nikon). A 40 X water-immersion objective lens (MRD07420, Nikon; NA = 0.8, working distance 3.5 mm) was used to image individual taste buds, and multi-plane images were acquired using NIS-Elements software AR version 5.21.03 (Nikon Instruments Inc., Melville, NY, USA, 64-bit) and an objective z-piezo stage connected to a controller (MCL NanoDrive, MCL, Madison, WI, USA).

### 2.3. Data Analysis

#### 2.3.1. Study 1: Psychophysics Data Analysis

To determine whether habitual LCS consumption was associated with a reduced perception of sweet intensity, we conducted separate mixed ANOVAs for each study visit (Glucose and Fructose visit). These analyses included “group” (HC and NHC) as the between-subject factor, and “solution” (sucralose, sugar (GLU 800 or FRU 475), and binary blends) as the within-subject factor. The data from GLU 111 and FRU 45 solutions were excluded from this analysis because their perceived intensities were extremely low (ranging from “no sensation” to “barely detectable”), resulting in restricted variability and violating the assumption of homogeneity of variance when compared with the more concentrated sweetener solutions. The mean perceived intensities of these solutions by group are presented separately. Data sets for taste intensity ratings were positively skewed and required log transformations (log (x + 1)) to approximate a normal distribution.

To determine whether habitual LCS consumption was associated with faster or to a greater degree sweet taste adaptation to the sweetness of sugars, sucralose, or its blends, we conducted separate mixed ANOVAs for each study visit (Glucose, Fructose, and Last visit). The main outcome variable was the percentage degree of adaptation of each trial versus trial 1. That is, to calculate the percentage degree of adaptation, the percentage decrease in perceived sweet taste intensity of adapting solutions (trials 2–10) relative to the perceived sweet taste intensity of solution 1 in each test was calculated. A mixed ANOVA was conducted for adapting solutions (i.e., trials 2–9 for Glucose and Fructose visits, and trial 2–10 for Last visit). We included “adapting condition” (sucralose and binary blend) and “trials” (2–9 or 2–10 for the Last visit) as the within-subject factors and “group” (HC and NHC) as the between-subject factor. We also analyzed whether there were differences in the taste perception of sugar solutions (GLU 800 and FRU 475) after adapting to sucralose alone or to its binary blends (i.e., trial 1 vs. trial 10) (i.e., cross-adaptation or the opposite phenomenon of cross-sensitization) and whether this was different between HC and NHC. For these analyses, we used separate mixed ANOVAs for each visit with “group” (HC and NHC) as the categorical factor and “trial” as the within-subjects factor.

Because the HC group had a higher weight than the NHC group (*p* = 0.005), we repeated the mixed ANOVAs with weight as a covariate (ANCOVA); however, since results remained unchanged, we are reporting here the results of the ANOVAs. Supplementary Tables S1 and S2 present the results of these two approaches (i.e., mixed ANOVA vs. mixed ANCOVA). To reduce multicollinearity in the model, which included several interactions, and to allow easier interpretation of the results, we centered the variable weight to use it in the ANCOVA. Significant interactions were further analyzed using Fisher’s least significant difference tests. Analyses were performed in Statistica 14.0 (TIBCO Software, CA, USA), and *p*-values were adjusted for multiple comparisons using the Benjamini–Hochberg false discovery rate method [29].

#### 2.3.2. Study 2: Molecular Data Analysis

Calcium traces obtained from in vivo calcium imaging data were analyzed based on MATLAB functions and open-source algorithms. Initially, images were motion corrected by utilizing a non-rigid motion correction algorithm (NoRMCorre) in MATLAB R2024b (MathWorks, Natick, MA, USA) [30,31]. Subsequently, the calcium traces were converted to ∆F/F by calculating the change in fluorescence at specific selected regions of interest. ImageJ (version 1.51n, National Institutes of Health) or Adobe Photoshop version 26.11 (Adobe Inc., San Jose, CA, USA) was utilized for visualizing and linearly adjusting brightness and contrast.

Parameters for analyses, such as the peak of ∆F/F, were obtained using MATLAB R2024b. Statistical analysis was performed using Prism 9 software (GraphPad) and a statistical analysis package in Python version 3.10.8 (Python software Foundation, 2022). To assess the significance of responses in subsequent trials relative to the first trial, a repeated measures ANOVA with Geisser–Greenhouse correction was applied. Linear fitting was applied to derive the linear regression trendline, and the slope were statistically compared using an unpaired *t*-test (Mann–Whitney).

## 3. Results

### 3.1. Study 1: Human Psychophysics Study Results

#### 3.1.1. Subject Characteristics

Of the 100 recruited participants, seven were lost to follow-up after screening, seven did not follow sensory protocol instructions, and five were found to be screening failures (detailed reasons for exclusion are shown in Figure 1). Therefore, a total of 81 participants completed at least one of the sensory tests and were included in the data analysis. Table 2 compares participant characteristics between the HC and NHC.

#### 3.1.2. Taste Intensity Test Results

**Figure 3:**
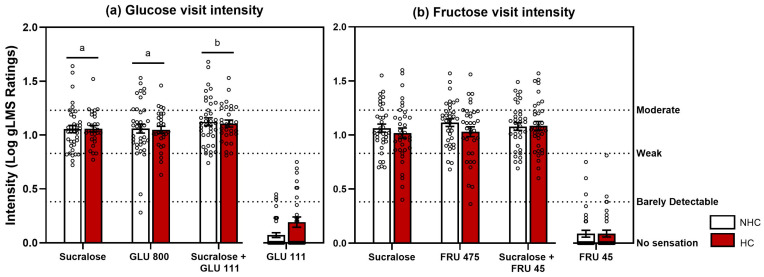
A negligible amount of the glucose (111 mM) but not fructose (45 mM) enhances the sweetness of sucralose in HC and NHC. (**a**) Glucose visit taste intensity ratings. Solutions include sucralose 0.6 mM, GLU 111, GLU 800, and sucralose + GLU 111 (*n* = 67). (**b**) Fructose visit intensity ratings. Solutions include sucralose 0.6 mM, FRU 45, FRU 475, and sucralose + FRU 45 (*n* = 69). The right axis displays the verbal descriptors shown to participants when using the gLMS; numerical values are not visible to them. Data are mean values ± SEM, with individual data points shown for each group. Solutions that do not share a letter differed at post hoc testing (*p* < 0.05).

There were no significant differences in sweetness ratings between the HC and NHC groups (*p*-values > 0.6 for both Glucose, *n*: NHC = 38, HC = 29 and Fructose visits, *n*: NHC = 35, HC = 34), and no significant group x solution interactions (Glucose visit: *p* = 0.9, Fructose visit: *p* = 0.1) (Figure 3). Sucralose was rated as equi-sweet to both GLU 800 and FRU 475. Although GLU 111 was barely detectable on its own, it significantly enhanced the perceived sweetness of sucralose when combined in a mixture (*p* = 0.007). In contrast, adding FRU 45 to sucralose did not result in a significant increase in perceived sweetness (*p* = 0.1). Details of statistical results can be found in Supplementary Tables S1 and S2.

#### 3.1.3. Sweet Taste Adaptation Results

**Figure 4:**
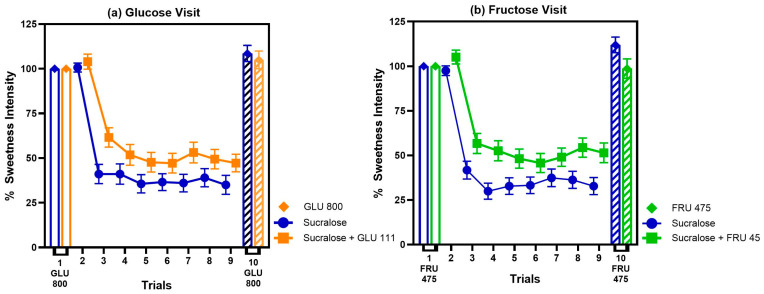
A barely detectable amount of sugar blended with sucralose reduces the degree of adaptation to sucralose compared to when adapting to sucralose alone. (**a**) Glucose visit: *p* value for solution = 0.003 and trial < 0.0001, *n* = 67). (**b**) Fructose visit; *p* value for solution < 0.0001 and trial < 0.0001; *n* = 69. Values are the percent change in mean sweetness intensity relative to trial 1 ± SEM.

Both HC and NHC participants showed a similar degree of sweet taste adaptation when repeatedly tasting solutions of GLU 800, FRU 475, sucralose, and binary blends of sucralose with a sugar (all *p* values > 0.5; see Supplementary Figure S1). However, the presence of a GLU 111 or FRU 45 in binary blends significantly reduced the degree of adaptation to sucralose compared to when sucralose was presented alone. This “adapting condition” effect is illustrated in Figure 4, which shows that across trials there was a significant main effect of solution for both study visits (*p* < 0.003). Details of statistical results can be found in Supplementary Tables S1 and S2.

**Figure 5:**
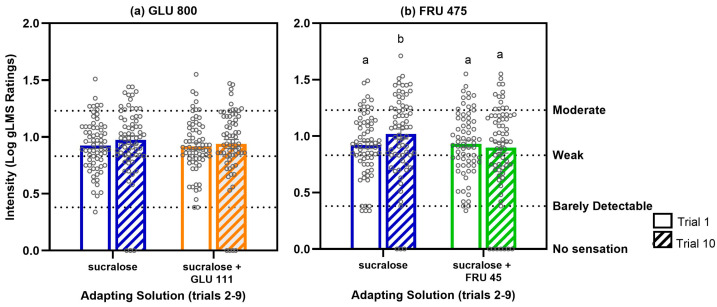
Adaptation to sucralose increases the sweetness perception of FRU 475, but not GLU 800 and adding FRU 45 mM to the adapting sucralose solution prevents this effect. (**a**) Glucose visit adaptation tests where trials 1 and 10 were GLU 800 (solution x trial interaction: *p* = 0.8, *n* = 67). (**b**) Fructose visit adaptation tests where trials 1 and 10 were FRU 475 (solution x trial interaction: *p* = 0.02, *n* = 69). The right axis displays the verbal descriptors shown to participants when using the gLMS; numerical values are not visible to them. Data are mean values ± SEM, with individual data points shown for each group. Trials that do not share a letter differed at post hoc testing (*p* < 0.05).

Following adaptation to sucralose alone, participants reported a significant increase in the perceived sweetness of FRU 475 in trial 10 compared to trial 1. This effect was not observed after adaptation to the sucralose + FRU 45 blend (solution x trial interaction: *p* = 0.02; Figure 5). In contrast, during the Glucose visit, sweetness ratings for trial 10 did not differ from trial 1 under either adapting condition (solution x trial interaction: *p* = 0.8; Figure 5), indicating no measurable cross-adaptation effect.

**Figure 6:**
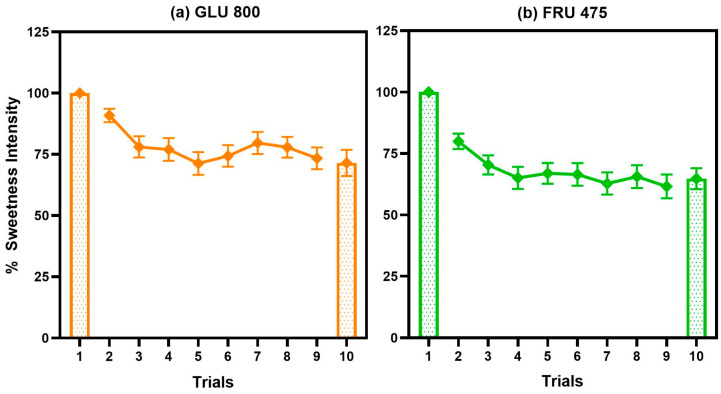
Participants exhibited significant adaptation to repeated exposures of both pure sugar solutions (GLU 800 and FRU 475). (**a**) Adaptation to GLU 800 (*n* = 55). (**b**) Adaptation to FRU 475 (*n* = 58). All *p* < 0.003 for trials. Values are the percent change in mean sweetness intensity relative to trial 1 ± SEM.

During the last visit, participants from both LCS groups showed similar patterns of adaptation to repeated tasting of pure sugars (main effect of trial for both GLU 800 and FRU 475, *p*-values < 0.003 for each, no effect of LCS group or interactions, all *p*-values > 0.6; Figure 6). However, the magnitude of adaptation to these sugars was visibly lower than that observed for an equi-sweet concentration of sucralose. Specifically, relative to the first trial, adaptation reached 73% ± 4% for glucose, 62% ± 5% for fructose (Figure 6), and on average 34% ± 5% for sucralose (Figure 4).

#### 3.1.4. In Vivo Imaging of Taste Buds When Adapting to Sucralose and Its Mixtures with Sugars

**Figure 7:**
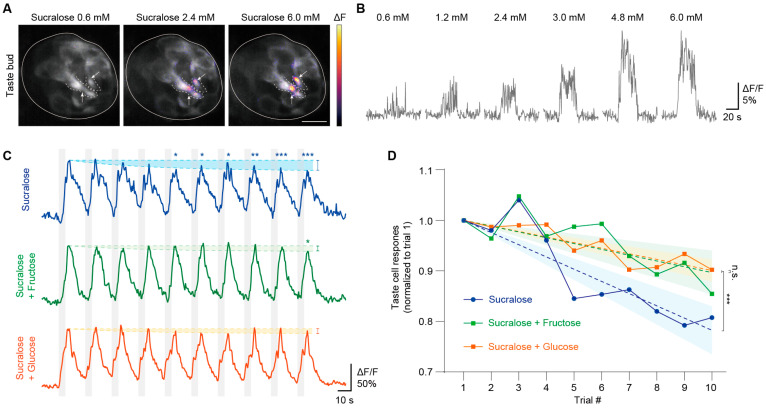
In vivo taste bud calcium responses during adaptation to sucralose and its mixtures with diluted sugars. (**A**) Representative image of concentration-dependent calcium responses of sweet-sensing taste cells. The white line indicates the boundary of a fungiform papilla taste bud in a top view. The white arrows indicate the response area within the apical regions of taste-responsive cells (outlined by the white dashed line). (Scale bar = 10 μm.) (**B**) Calcium traces showing concentration-dependent responses in sweet-sensing taste cells. Stronger calcium responses were observed with increasing sucralose concentrations, with robust calcium activity becoming evident at levels of 2.4 mM or higher. (**C**) Average trace of calcium responses of sweet cells to 2.4 mM sucralose (*n* = 22 cells), 2.4 mM sucralose with FRU 45, (*n* = 22 cells) and 2.4 mM sucralose with GLU 111 (*n* = 17 cells): Adding fructose or glucose in binary blends reduced the degree of adaptation to sucralose compared to sucralose alone (* *p* <0.05, ** *p* < 0.01, *** *p* < 0.001 by repeated measures ANOVA compared to trial 1). (**D**) Simple linear regression lines with 95% confidence intervals of calcium responses: blends of sucralose with sugar showed a smaller decay slope than sucralose alone (*** *p* < 0.001, by unpaired *t* test). n.s. denotes no significant difference between Sucralose + Fructose and Sucralose + Glucose.

Previous studies have shown that distinct sweet molecules can activate divergent signaling pathways in taste cells [32,33]. Building on this, we sought to determine whether the adaptation patterns observed in human sensory experiments, particularly the trend of adaptation, originate within the peripheral taste system. To this end, we employed in vivo imaging of taste cells of mice under conditions designed to closely parallel human sensory paradigms. Sucralose concentrations over 2.4 mM elicited a sustained elevation in calcium response (Figure 7A,B). The repetitive stimulation of taste buds with 2.4 mM sucralose and its mixture with sugars resulted in decreased calcium responses (*p* < 0.05; Figure 7C). Compared to sucralose alone, the inclusion of diluted concentrations of fructose or glucose in binary blends reduced the degree of adaptation to sucralose (*p* < 0.001; Figure 7D).

## 4. Discussion

This study examined both perceptual and molecular aspects of sweet taste adaptation to the high potency LCS, sucralose, assessed alone and in binary blends with barely detectable concentrations of glucose (111 mM) or fructose (45 mM). The human study assessed whether habitual LCS consumption was associated with reduced perceived sweetness intensity or greater adaptation across repeated exposures. The complementary mouse model evaluated intrinsic taste cell responses to similar solutions to provide mechanistic insights into adaptation. Contrary to our hypothesis, no significant differences in sweetness perception or adaptation were observed between HCs and NHCs, suggesting that habitual use alone may not account for individual variability in sweet-taste adaptation. Across all participants, repeated exposure to sucralose alone produced stronger adaptation than when sucralose was presented in blends with GLU 111 or FRU 45. A similar pattern was observed in the molecular assays, where we found that the degree of adaptation differed significantly between sucralose alone and its sugar blends, indicating that mechanisms within taste cells directly contribute to the human sensory outcomes.

These findings are consistent with prior studies that combined an LCS with sugar to enhance perceived sweetness and reduce adaptation [15,16,34,35,36,37]. However, our study extends previous work by testing blends at concentrations representative of commercially available products. Earlier studies often examined binary mixtures in which each component was equi-sweet to 3–8% sucrose, typically combined in a 50:50 sweeter-to-sweetener ratio, and synergy frequently diminished at equi-sweet solutions that were higher than 8% sucrose concentrations [36]. For example, Schiffman (2003) evaluated binary blends of sucralose, fructose, and glucose at intensities equi-sweet to 5% sucrose, quantifying the synergy by comparing summed single-component intensities to self-mixtures [16]. In contrast, we used sucralose at 0.6 mM (approximately equi-sweet to ~10% sucrose, comparable to many beverages; [38]) blended with GLU 111 or FRU 45, reflecting the composition of commercial sweetener packets.

Although GLU 111 or FRU 45 alone were generally rated at or below “barely detectable,” their inclusion in blends with sucralose attenuated adaptation compared to sucralose alone. Because the sugars were detectable but of very low intensity, traditional additive or self-mixture synergy calculations were not feasible. Nonetheless, the higher perceived sweetness and reduced adaptation in the blends suggest a subtle synergistic effect. These psychophysics responses were consistent with molecular findings in our mouse model, as GLU 111 or FRU 45 modulated receptor calcium signaling and reduced adaptation. Practically, these results suggest that small amounts of sugars often included in commercial sweetener packets as bulking agents may not be perceptually obvious alone but can subtly influence taste dynamics and adaptation, a consideration for both product formulation and sensory-consumer research.

Our findings also provide insight into cross-adaptation, defined as a decrease in responsiveness to one stimulus following adaptation to a different stimulus [11]. Previous studies have reported stronger cross-adaptation effects after exposure to sugars than to LCSs [11,12,39,40]. In our study, repeated exposure to high concentrations of GLU 800 and FRU 475 reduced subsequent sweetness ratings for those same sugars at trial 10, reflecting self-adaptation (Figure 6). However, ratings for GLU 800 remained unchanged from trial 1 to trial 10, after adaptation to either sucralose alone or in a blend, indicating the absence of cross-adaptation. Notably, ratings for FRU 475 increased after adaptation to sucralose alone but remained stable after adaptation to the sucralose–fructose blend. This pattern suggests that a small degree of self-adaptation to the FRU 45 present in the blend may have blunted the apparent cross-sensitization effect. These findings are consistent with prior evidence that adaptation dynamics are sweetener-specific and likely reflect differences in how sucralose and caloric sugars engage and desensitize the T1R2 + T1R3 receptor complex [1]. Although psychophysical data have limitations for inferring molecular mechanisms of taste perception, our observation of cross-sensitization with fructose suggests that, following adaptation, sucralose may influence receptor conformation in a manner resembling positive allosteric modulation for fructose. However, the effect was very subtle. Further research using cell-based assays, computational docking, and structural biology approaches is needed to better understand the complex ligand–receptor interactions that underlie the pharmacology of the human sweet taste receptor.

This study has several limitations. First, we tested only one LCS (sucralose), which limits the generalizability of our findings to other LCSs. Second, body weight differed between HC and NHC participants, with the HCs being heavier than NHCs. This difference is expected since individuals with higher BMI are more likely to consume LCS products [41]. However, including body weight as a covariate did not alter the results. Third, we did not collect detailed data on participants’ daily sugar intake, which could influence adaptation patterns (for example, by grouping them in overall sweetness exposure). Fourth, the rodent calcium imaging experiments required the use of higher sucralose concentrations (2.4 mM) than the human studies (0.6 mM). These differences in concentrations arise because there is an inherent gap between the concentrations required to elicit reliable measurable taste cell responses and those sufficient to generate perceptual reports in behavioral assays. For example, in behavioral studies of sucrose (e.g., preference or intake assays) in rodents, ~1% solutions (~30 mM) are most frequently used, with more than 70% of studies adopting this concentration [42,43]. In contrast, imaging of the geniculate ganglion (ganglion of taste bud afferent nerves) shows an EC_50_ of ~137 mM [44], and experiments are typically conducted in the range of 100–300 mM (about 1.5–2 × EC_50_; the response ratio at 30 mM does not exceed 15%). Similarly, behavioral thresholds for citric acid (~1–5 mM) or NaCl (~75 mM) are consistently lower than the concentrations used in chorda tympani or geniculate ganglion recordings (7–10 mM and ~150 mM, respectively) [44,45,46]. These examples suggest that the concentration needed to reliably elicit calcium signals from individual taste cells may be higher than those required for perceptual detection, possibly because behavioral responses reflect integrated input from multiple sensory cells and nerves, whereas imaging captures activity at the single-cell level. Despite these differences, attenuated adaptation was consistently observed at higher sucralose concentrations when small amounts of sugars were added in our rodent experiment. This indicates that sugar-induced modulation of taste cell responses to sucralose is reliably present in mice. Therefore, the discrepancies in sucralose concentration between humans and mice do not undermine the translational relevance of our findings but instead point to peripheral taste bud signaling systems as the likely source. Finally, genetic variation in sweet taste receptors and their transductive pathways was not assessed and represents an important area for future research.

## 5. Conclusions

In conclusion, this study integrates human psychophysical data with molecular evidence from taste buds in rodents. Although habitual LCS consumption did not associate with alterations in sweetness perception or adaptation for glucose, fructose, sucralose, or the sucralose blends, we here show that barely detectable levels of sugar in sucralose blends can attenuate sweet taste adaptation both in humans and a rodent model. The complementary mouse model provides mechanistic support that sweetener composition modulates sweetness perception at the peripheral level. Together, these findings extend prior work by linking perceptual and receptor-level responses and highlighting considerations for the design of LCS formulations. Future studies may explore differences in the sweetness perception of other LCSs and blends in habitual and non-habitual LCS consumers.

## Figures and Tables

**Table 1:** Sensory stimuli per study visit.

Solution	Abbreviation	Glucose Visit		Fructose Visit		Last Visit
		Taste Intensity	Adaptation	Taste Intensity	Adaptation	Adaptation
Sucralose 0.6 mM	SCL	+	+	+	+	-
Glucose 111 mM	GLU 111	+	-	-	-	-
Glucose 800 mM	GLU 800	+	+	-	-	+
Sucralose blend G	SCL + GLU 111	+	+	-	-	-
Fructose 45 mM	FRU 45	-	-	+	-	-
Fructose 475 mM	FRU 475	-	-	+	+	+
Sucralose blend F	SCL + FRU 45	-	-	+	+	-

**Table 2:** Participant Characteristics.

	Glucose Visit (*n* = 67)			Fructose Visit (*n* = 69)			Last Visit (*n* = 60)		
	NHC (*n* = 38)	HC (*n* = 29)	*p*-Value	NHC (*n* = 35)	HC (*n* = 34)	*p*-Value	NHC (*n* = 31)	HC (*n* = 29)	*p*-Value
Sex, *n*									
Male	16	13	0.8	15	17	0.5	13	15	0.6
Female	22	16		20	17		18	14	
Race, *n*									
White	23	22	0.3	23	26	0.2	18	23	0.08
Asian	11	3		10	3		10	2	
Black	3	3		2	3		3	3	
Mixed race	1	1		-	1		-	1	
Did not Specify	-	-		-	1		-	-	
Age, years	28.0 (21.0–34.0)	31.0 (25.0–35.0)	0.1	28.0 (21.0–36.0)	31.5 (26.0–41.0)	0.1	28.0 (21.0–37.0)	32.0 (28.0–41.0)	0.2
Weight, kg	73.4 (20.0)	86.9 (17.3)	**0.005**	73.3 (19.9)	88.1 (15.9)	**0.001**	74.1 (20.6)	88.5 (17.1)	**0.005**
Height, m	1.69 (0.08)	1.70 (0.1)	0.7	1.70 (0.08)	1.72 (0.09)	0.4	1.69 (0.09)	1.71 (0.1)	0.4
BMI, kg/m^2^	25.6 (6.9)	30.0 (5.9)	**0.007**	25.4 (6.6)	29.9 (5.8)	**0.003**	25.8 (6.8)	30.3 (6.0)	**0.009**

Values are presented as mean (SD), except age, which was not normally distributed and is presented as median (semi-interquartile range). Values in bold denote significant differences between groups.

## Data Availability

The data presented in this study are available upon request from the corresponding authors for both studies. The data are not publicly available in accordance with the consent provided by participants on the use of confidential data.

## Supplementary Materials

The following supporting information can be downloaded at: https://www.mdpi.com/article/10.3390/nu17193075/s1, Table S1: Human sensory study statistics; Table S2: Human sensory study results with centered weight as covariate; Figure S1: Similar degree of adaptation to sweetness in both HC and NHC.

## Abbreviations

The following abbreviations are used in this manuscript:

LCS	Low-Calorie Sweetener
HC	Habitual Low-Calorie Sweetener Consumers
NHC	Non-Habitual Low-Calorie Sweetener Consumers
GLU	Glucose
FRU	Fructose
GCPR	G-Coupled protein receptors
gLMS	General Labeled Magnitude Scale

## References

[1] Juen Z., Lu Z., Yu R., Chang A.N., Wang B., Fitzpatrick A.W.P., Zuker C.S. (2025). The Structure of Human Sweetness. Cell.

[2] Nie Y., Vigues S., Hobbs J.R., Conn G.L., Munger S.D. (2005). Distinct Contributions of T1R2 and T1R3 Taste Receptor Subunits to the Detection of Sweet Stimuli. Curr. Biol..

[3] Hao S., Guthrie B., Kim S.K., Balanda S., Kubicek J., Murtaza B., Khan N.A., Khakbaz P., Su J., Goddard W.A. (2024). Steviol Rebaudiosides Bind to Four Different Sites of the Human Sweet Taste Receptor (T1R2/T1R3) Complex Explaining Confusing Experiments. Commun. Chem..

[4] Servant G., Tachdjian C., Tang X.Q., Werner S., Zhang F., Li X., Kamdar P., Petrovic G., Ditschun T., Java A. (2010). Positive Allosteric Modulators of the Human Sweet Taste Receptor Enhance Sweet Taste. Proc. Natl. Acad. Sci. USA.

[5] Kawasaki M., Kidera Y., Goda R., Taketani C., Ide M., Fujii W., Nakagita T., Misaka T. (2025). Distinct Potency of Compounds Targeting the T1R3 Subunit in Modulating the Response of Human Sweet and Umami Taste Receptors. Sci. Rep..

[6] Wark B., Lundstrom B.N., Fairhall A. (2007). Sensory Adaptation. Curr. Opin. Neurobiol..

[7] Low Y.Q., Lacy K., Keast R. (2014). The Role of Sweet Taste in Satiation and Satiety. Nutrients.

[8] Dubose C.N., Meiselman H.L. (1979). Note: Individual Differences in Taste Adaptation. Chem. Senses.

[9] Theunissen M.J.M., Kroeze J.H.A., Schifferstein H.N.J. (2000). Method of Stimulation, Mouth Movements, Concentration, and Viscosity: Effects on the Degree of Taste Adaptation. Percept. Psychophys..

[10] Green B.G., Nachtigal D. (2015). Temperature Affects Human Sweet Taste via At Least Two Mechanisms. Chem. Senses.

[11] Hewson L., Tarrega A. (2017). Sensory Adaptation. Time-Dependent Measures of Perception in Sensory Evaluation.

[12] Schiffman S.S., Cahn H., Lindley M.G. (1981). Multiple Receptor Sites Mediate Sweetness: Evidence from Cross Adaptation. Pharmacol. Biochem. Behav..

[13] Rodriguez Y.A., Roebber J.K., Dvoryanchikov G., Makhoul V., Roper S.D., Chaudhari N. (2021). “Tripartite Synapses” in Taste Buds: A Role for Type I Glial-like Taste Cells. J. Neurosci..

[14] Park G.Y., Lee G., Yoon J., Han J., Choi P., Kim M., Lee S., Park C., Wu Z., Li Y. (2025). Glia-like Taste Cells Mediate an Intercellular Mode of Peripheral Sweet Adaptation. Cell.

[15] Schiffman S.S., Booth B.J., Carr B.T., Losee M.L., Sattely-Miller E.A., Graham B.G. (1995). Investigation of Synergism in Binary Mixtures of Sweeteners. Brain Res. Bull..

[16] Schiffman S.S., Sattely-Miller E.A., Graham B.G., Zervakis J., Butchko H.H., Stargel W.W. (2003). Effect of Repeated Presentation on Sweetness Intensity of Binary and Ternary Mixtures of Sweeteners. Chem. Senses.

[17] Schiffman S.S., Sattely-Miller E.A., Bishay I.E. (2007). Time to Maximum Sweetness Intensity of Binary and Ternary Blends of Sweeteners. Food Qual. Prefer..

[18] Bornstein B.L., Wiet S.G., Pombo M. (1993). Sweetness Adaptation of Some Carbohydrate and High Potency Sweeteners. J. Food Sci..

[19] Myers E.A., Passaro E.M., Hedrick V.E. (2018). The Reproducibility and Comparative Validity of a Non-Nutritive Sweetener Food Frequency Questionnaire. Nutrients.

[20] Pepino M.Y., Mennella J.A. (2007). Effects of Cigarette Smoking and Family History of Alcoholism on Sweet Taste Perception and Food Cravings in Women. Alcohol. Clin. Exp. Res..

[21] Pepino M.Y., Bradley D., Eagon J.C., Sullivan S., Abumrad N.A., Klein S. (2014). Changes in Taste Perception and Eating Behavior after Bariatric Surgery-Induced Weight Loss in Women. Obesity.

[22] Wasalathanthri S., Hettiarachchi P., Prathapan S. (2014). Sweet Taste Sensitivity in Pre-Diabetics, Diabetics and Normoglycemic Controls: A Comparative Cross Sectional Study. BMC Endocr. Disord..

[23] Doty R.L., Shaman P., Dann M. (1984). Development of the University of Pennsylvania Smell Identification Test: A Standardized Microencapsulated Test of Olfactory Function. Physiol. Behav..

[24] Bartoshuk L.M., Duffy V.B., Green B.G., Hoffman H.J., Ko C.W., Lucchina L.A., Marks L.E., Snyder D.J., Weiffenbach J.M. (2004). Valid Across-Group Comparisons with Labeled Scales: The GLMS versus Magnitude Matching. Physiol. Behav..

[25] Romo-Romo A., Aguilar-Salinas C.A., Brito-Córdova G.X., Gómez-Díaz R.A., Almeda-Valdes P. (2018). Sucralose Decreases Insulin Sensitivity in Healthy Subjects: A Randomized Controlled Trial. Am. J. Clin. Nutr..

[26] De Graaf C., Frijters J.E.R., Van Trijp H.C.M. (1987). Taste Interaction between Glucose and Fructose Assessed by Functional Measurement. Percept. Psychophys..

[27] Breslin P.A.S., Beauchamp G.K., Pugh E.N. (1996). Monogeusia for Fructose, Glucose, Sucrose, and Maltose. Percept. Psychophys..

[28] Han J., Choi M. (2018). Comprehensive Functional Screening of Taste Sensation in Vivo. bioRxiv.

[29] Benjamini Y., Hochberg Y. (1995). Controlling the False Discovery Rate: A Practical and Powerful Approach to Multiple Testing. J. R. Stat. Soc. Ser. B Stat. Methodol..

[30] Pnevmatikakis E.A., Giovannucci A. (2017). NoRMCorre: An Online Algorithm for Piecewise Rigid Motion Correction of Calcium Imaging Data. J. Neurosci. Methods.

[31] Thurman S.T., Guizar-Sicairos M., Fienup J.R. (2008). Efficient Subpixel Image Registration Algorithms. Opt. Lett..

[32] Breslin P.A.S., Izumi A., Tharp A., Ohkuri T., Yokoo Y., Flammer L.J., Rawson N.E., Margolskee R.F. (2021). Evidence That Human Oral Glucose Detection Involves a Sweet Taste Pathway and a Glucose Transporter Pathway. PLoS ONE.

[33] von Molitor E., Riedel K., Krohn M., Hafner M., Rudolf R., Cesetti T. (2021). Sweet Taste Is Complex: Signaling Cascades and Circuits Involved in Sweet Sensation. Front. Hum. Neurosci..

[34] Ayya N., Lawless H.T. (1992). Quantitative and Qualitative Evaluation of High-Intensity Sweeteners and Sweetener Mixtures. Chem. Senses.

[35] Tan V.W.K., Wee M.S.M., Tomic O., Forde C.G. (2019). Temporal Sweetness and Side Tastes Profiles of 16 Sweeteners Using Temporal Check-All-That-Apply (TCATA). Food Res. Int..

[36] Wang C., Liu Y., Zhao X., Liu B. (2022). Current Advances and Future Aspects of Sweetener Synergy: Properties, Evaluation Methods and Molecular Mechanisms. Appl. Sci..

[37] Mora M., Wijaya F., Jiang G., Gibney P., Dando R. (2023). Sensory Profiling of Natural Sweeteners and Sucrose–Sweetener Binary Mixtures. J. Food Sci..

[38] Ventura E.E., Davis J.N., Goran M.I. (2011). Sugar Content of Popular Sweetened Beverages Based on Objective Laboratory Analysis: Focus on Fructose Content. Obesity.

[39] Schiffman S.S., Pecore S.D., Booth B.J., Losee M.L., Carr B.T., Sattely-Miller E., Graham B.G., Warwick Z.S. (1994). Adaptation of Sweeteners in Water and in Tannic Acid Solutions. Physiol. Behav..

[40] Mcburney D.H. (1972). Gustatory Cross Adaptation between Sweet-Tasting Compounds. Percept. Psychophys..

[41] Sylvetsky A.C., Rother K.I. (2016). Trends in the Consumption of Low-Calorie Sweeteners. Physiol. Behav..

[42] Primo M.J., Fonseca-Rodrigues D., Almeida A., Teixeira P.M., Pinto-Ribeiro F. (2023). Sucrose Preference Test: A Systematic Review of Protocols for the Assessment of Anhedonia in Rodents. Eur. Neuropsychopharmacol..

[43] Verharen J.P.H., de Jong J.W., Zhu Y., Lammel S. (2023). A Computational Analysis of Mouse Behavior in the Sucrose Preference Test. Nat. Commun..

[44] Wu A., Dvoryanchikov G., Pereira E., Chaudhari N., Roper S.D. (2015). Breadth of Tuning in Taste Afferent Neurons Varies with Stimulus Strength. Nat. Commun..

[45] Tordoff M.G. (2007). Taste Solution Preferences of C57BL/6J and 129X1/SvJ Mice: Influence of Age, Sex, and Diet. Chem. Senses.

[46] Barretto R.P.J., Gillis-Smith S., Chandrashekar J., Yarmolinsky D.A., Schnitzer M.J., Ryba N.J.P., Zuker C.S. (2015). The Neural Representation of Taste Quality at the Periphery. Nature.

