# Supplementary material for: Sweet Taste Adaptation to Sugars, Sucralose, and Their Blends: A Human and Rodent Perspective
Source: Nutrients. 2025 Sep 27;17(19):3075. doi: 10.3390/nu17193075 (PMC12526075; doi:10.3390/nu17193075)
Supplement: Supplementary file 1 [file nutrients-17-03075-s001.zip › nutrients-3856455-supplementary.pdf]

Table S1. Human sensory study statistics

Taste Test	Effects	Glucose Visit (NHC = 38, HC = 29)			Fructose Visit (NHC = 35, HC = 34)			Last Visit GLU 800 (NHC = 30, HC = 25)			Last Visit FRU 475 (NHC = 30, HC = 28)		
		**d.f.**	**F**	**p-value***	**d.f**	**F**	**p-value**	**d.f.**	**F**	**p-value***	**d.f.**	**F**	**p-value***
**Intensity**	LCS Group	1, 65	0.033	0.9	1, 67	0.61	0.6						
	Solution	2, 130	7.17	**0.007**	2, 134	3.39	0.1						
	Solution x LCS group	2, 130	0.16	0.9	2, 134	3.67	0.1						
**GLU 111/FRU 45	LCS Group	1, 65	6.17	0.07	1, 67	0.00058	1.0						
**Adaptation**	LCS Group	1, 65	0.86	0.6	1, 67	0.081	0.9	1, 53	1.22	0.6	1, 56	1.05	0.6
	Solution	1, 65	14.60	**0.003**	1, 67	30.78	**0.00**						
	Solution x LCS Group	1, 65	1.28	0.6	1, 67	3.11	0.3						
	Trial	7, 455	57.98	**0.000**	7, 469	67.78	**0.00**	8, 424	0.29	**0.000**	8, 448	0.44	**0.003**
	Trial x LCS Group	7, 455	1.20	0.6	7, 469	1.00	0.7	8, 424	4.67	1.0	8, 448	3.59	1.0
	Solution x Trial	7, 455	1.40	0.6	7, 469	0.10	0.7						
	Solution x Trial x LCS Group	7, 455	0.70	0.9	7, 469	1.33	0.6						
**Trial 1 and 10**	LCS Group	1, 65	0.92	0.6	1, 67	0.071	0.9						
	Solution	1, 65	1.06	0.6	1, 67	6.29	0.07						
	Solution x LCS Group	1, 65	0.14	0.9	1, 67	0.19	0.9						
	Trial	1, 65	2.24	0.4	1, 67	1.01	0.6						
	Trial x LCS Group	1, 65	0.96	0.6	1, 67	0.0024	1.0						
	Solution x Trial	1, 65	0.38	0.8	1, 67	8.91	**0.02**						
	Solution x Trial x LCS Group	1, 65	0.039	0.9	1, 67	0.18	0.9						

*All p-values reported were adjusted for false discovery using the Benjamin-Hochberg correction. **Separate analysis for GLU 111 and FRU 45 intensity ratings. Degree of freedom for each effect and error are reported under the “d.f.” column

Table S2. Human sensory study results with centered weight as covariate

Test	Effects	Glucose Visit (NHC = 38, HC = 29)			Fructose Visit (NHC = 35, HC = 34)			Last Visit GLU 800 (NHC = 30, HC = 25)			Last Visit FRU 475 (NHC = 30, HC = 28)		
		**d.f.**	**F**	**p-value**	**d.f**	**F**	**p-value**	**d.f.**	**F**	**p-value**	**d.f.**	**F**	**p-value**
**Taste Intensity**	Centered Weight	1, 64	1.36	0.5	1, 66	0.50	0.6						
	LCS Group	1, 64	0.051	0.6	1, 66	0.20	0.6						
	Solution	2, 128	7.05	**0.007**	2, 132	3.17	0.2						
	Solution x Centered Weight	2, 128	0.45	**0.6**	2, 132	0.81	0.6						
	Solution x LCS group	2, 128	0.35	0.6	2, 132	3.70	0.1						
**GLU 111/FRU 45	Centered Weight	1, 64	2.42	0.4	1, 66	0.068	0.6						
	LCS Group	1, 64	8.35	**0.03**	1, 66	0.0148	0.6						
**Adaptation trials (2-9) or (2-10)**	Centered Weight	1, 64	1.86	0.4	1, 66	0.39	0.6	1, 52	1.52	0.5	1, 55	0.014	0.6
	LCS Group	1, 64	0.17	0.6	1, 66	0.0004	0.6	1, 52	0.36	0.6	1, 55	0.97	0.5
	Solution	1, 64	14.22	**0.002**	1, 66	32.25	**0.000**						
	Solution x Centered Weight	1, 64	0.50	0.6	1, 66	1.97	0.4						
	Solution x LCS Group	1, 64	0.67	0.6	1, 66	31.22	0.5						
	Trial	7, 448	58.35	**0.000**	7, 462	67.21	**0.000**	8, 416	4.63	**0.000**	8, 440	3.85	**0.002**
	Trial x Centered Weight	7, 448	1.00	0.6	7, 462	1.37	0.4	8, 416	0.29	0.6	8, 440	0.58	0.6
	Trial x LCS Group	7, 448	0.79	0.6	7, 462	0.86	0.6	8, 416	0.307	0.6	8, 440	0.46	0.6
	Solution x Trial	7, 448	1.37	0.4	7, 462	1.00	0.6						
	Solution x Trial x Centered Weight	7, 448	1.13	0.5	7, 462	0.55	0.6						
	Solution x Trial x LCS Group	7, 448	0.49	0.6	7, 462	0.73	0.6						
**Trial 1 and 10**	Centered Weight	1, 64	3.01	0.3	1, 66	0.05	0.06						
	LCS Group	1, 64	2.26	0.4	1, 66	0.025	0.6						
	Solution	1, 64	1.03	0.5	1, 66	6.235	0.6						
	Solution x Centered Weight	1, 64	0.005	0.6	1, 66	0.19	0.6						
	Solution x LCS Group	1, 64	0.13	0.6	1, 66	0.53	0.6						
	Trial	1, 64	2.23	0.4	1, 66	0.92	0.5						
	Trial x Centered Weight	1, 64	0.045	0.6	1, 66	1.44	0.4						
	Trial x LCS Group	1, 64	0.97	0.5	1, 66	0.26	0.6						

Solution x Trial	1, 64	0.37	0.6	1, 66	8.52	**0.03**
Solution x Trial x Centered Weight	1, 64	0.001	0.6	1, 66	0.30	0.6
Solution x Trial x LCS Group	1, 64	0.029	0.6	1, 66	0.36	0.6

*All p-values reported were adjusted for false discovery using the Benjamin-Hochberg correction. **Separate analysis for GLU 111 and FRU 45 intensity ratings. Degree of freedom for each effect and error are reported under the "d.f." columns.

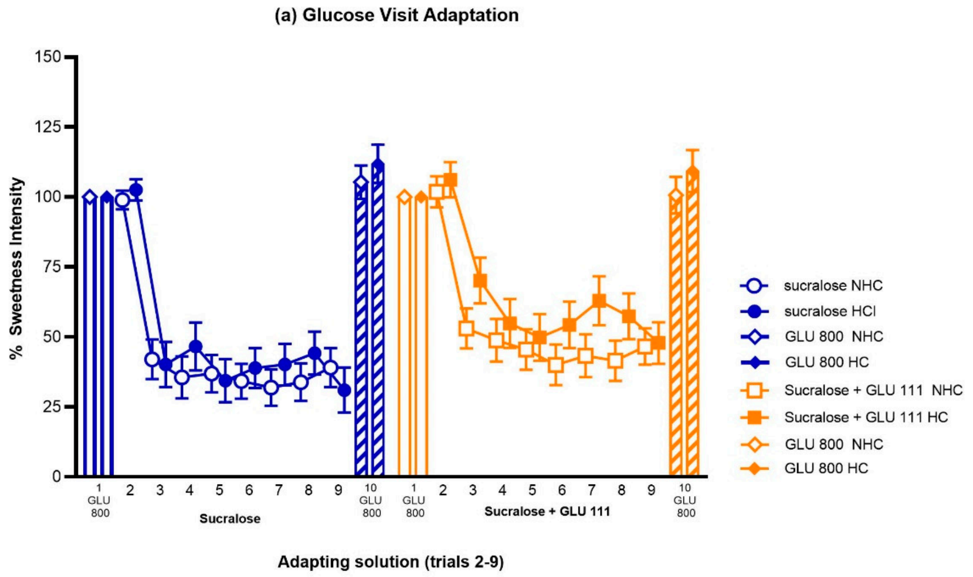
(a) Glucose Visit Adaptation
% Sweetness Intensity
150
125
100
75
50
25
0
1 GLU 800
2
3
4
5
6
7
8
9
10 GLU 800
1 GLU 800
2
3
4
5
6
7
8
9
10 GLU 800
Sucralose
Sucralose + GLU 111
Adapting solution (trials 2-9)
sucralose NHC
sucralose HCl
GLU 800 NHC
GLU 800 HC
Sucralose + GLU 111 NHC
Sucralose + GLU 111 HC
GLU 800 NHC
GLU 800 HC

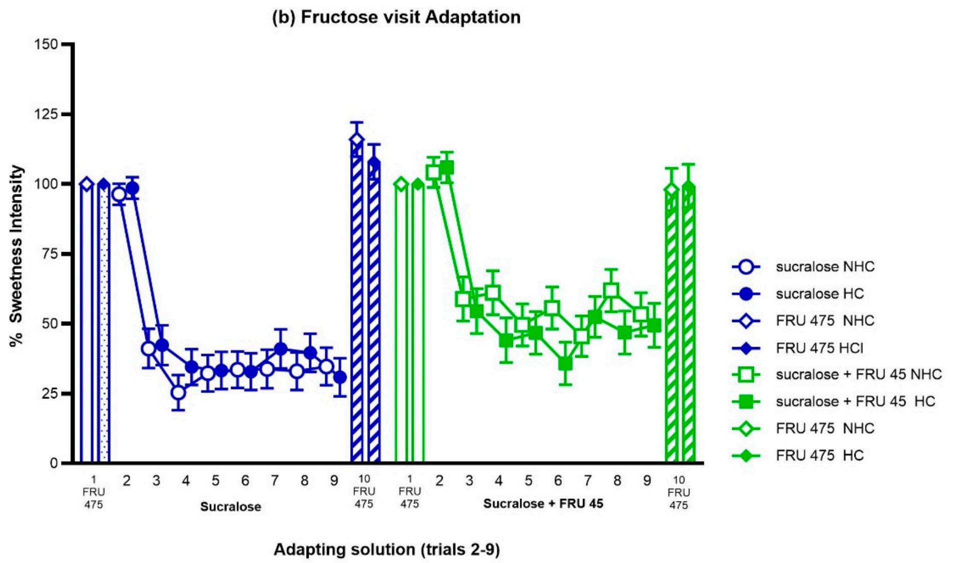
(b) Fructose visit Adaptation
% Sweetness Intensity
150
125
100
75
50
25
0
1 FRU 475
2
3
4
5
6
7
8
9
10 FRU 475
1 FRU 475
2
3
4
5
6
7
8
9
10 FRU 475
Sucralose
Sucralose + FRU 45
Adapting solution (trials 2-9)
sucralose NHC
sucralose HC
FRU 475 NHC
FRU 475 HCl
sucralose + FRU 45 NHC
sucralose + FRU 45 HC
FRU 475 NHC
FRU 475 HC

Figure S1: Similar degree of adaptation to sweetness in both HC and NHC (a) Glucose visit solutions; trail 1 and 10: glucose 800 mM, trial 2 to 9: sucralose 0.6 mM ± glucose 111 mM (p value; group = 0.5, condition by group by trial=0.5). n=38 NHC, n=29 HC (b) Fructose visit solutions; trail 1 and 10: fructose 475 mM, trial 2 to 9: sucralose 0.6 mM ± fructose 45 mM (p value; group = 0.8, condition by group by trial=0.6). n=35 NHC, n=34 HC. Values are percent change from mean of trail 1 ± SEM.
